# Tuning Ferulic Acid Solubility in Choline-Chloride- and Betaine-Based Deep Eutectic Solvents: Experimental Determination and Machine Learning Modeling

**DOI:** 10.3390/molecules29163841

**Published:** 2024-08-13

**Authors:** Tomasz Jeliński, Maciej Przybyłek, Rafał Różalski, Karolina Romanek, Daniel Wielewski, Piotr Cysewski

**Affiliations:** 1 Department of Physical Chemistry, Faculty of Pharmacy, Collegium Medicum in Bydgoszcz, Nicolaus Copernicus University in Toruń, Kurpińskiego 5, 85-096 Bydgoszcz, Poland; tomasz.jelinski@cm.umk.pl (T.J.); m.przybylek@cm.umk.pl (M.P.);; 2 Department of Clinical Biochemistry, Faculty of Pharmacy, Collegium Medicum in Bydgoszcz, Nicolaus Copernicus University in Toruń, Karłowicza 24, 85-950 Bydgoszcz, Poland; rafalr@cm.umk.pl

**Keywords:** ferulic acid, solubility, deep eutectic solvents, green solvents, molecular interactions, COSMO-RS, machine learning, support vector regressor

## Abstract

Deep eutectic solvents (DES) represent a promising class of green solvents, offering particular utility in the extraction and development of new formulations of natural compounds such as ferulic acid (FA). The experimental phase of the study undertook a systematic investigation of the solubility of FA in DES, comprising choline chloride or betaine as hydrogen bond acceptors and six different polyols as hydrogen bond donors. The results demonstrated that solvents based on choline chloride were more effective than those based on betaine. The optimal ratio of hydrogen bond acceptors to donors was found to be 1:2 molar. The addition of water to the DES resulted in a notable enhancement in the solubility of FA. Among the polyols tested, triethylene glycol was the most effective. Hence, DES composed of choline chloride and triethylene glycol (TEG) (1:2) with added water in a 0.3 molar ration is suggested as an efficient alternative to traditional organic solvents like DMSO. In the second part of this report, the affinities of FA in saturated solutions were computed for solute-solute and all solute-solvent pairs. It was found that self-association of FA leads to a cyclic structure of the $C_{2}^{8}$ type, common among carboxylic acids, which is the strongest type of FA affinity. On the other hand, among all hetero-molecular bi-complexes, the most stable is the FA-TEG pair, which is an interesting congruency with the high solubility of FA in TEG containing liquids. Finally, this work combined COSMO-RS modeling with machine learning for the development of a model predicting ferulic acid solubility in a wide range of solvents, including not only DES but also classical neat and binary mixtures. A machine learning protocol developed a highly accurate model for predicting FA solubility, significantly outperforming the COSMO-RS approach. Based on the obtained results, it is recommended to use the support vector regressor (SVR) for screening new dissolution media as it is not only accurate but also has sound generalization to new systems.

## 1. Introduction

Ferulic acid, a phenolic compound abundant in various plant tissues [1], has emerged as a captivating subject of scientific inquiry [2,3] and industrial interest [4,5], while as a component of traditional Chinese medicinal herbs, it has been used for centuries [6]. Chemically, it is a 4-hydroxy-3-methoxycinnamic acid, belonging to the wide group of phenolic acids. Its molecular structure is characterized by a phenolic ring with a hydroxyl group and a methoxy group, and can exist in both *cis* and *trans* forms [2]. Ferulic acid exhibits a plethora of biological activities that are the result of this unique structure. First of all, it is well known as an antioxidant agent [7,8]. Specifically, the hydroxyl and methoxy groups on the benzene ring are capable of donating hydrogen atoms to free radicals, thereby neutralizing their harmful effects and preventing oxidative damage to cells and tissues [9,10]. Additionally, ferulic acid can chelate metal ions involved in oxidative processes, further inhibiting the generation of reactive oxygen species (ROS) [11,12]. Secondly, ferulic acid exhibits anti-inflammatory properties through various mechanisms of action. One significant mechanism involves its ability to inhibit the activity of pro-inflammatory enzymes, such as cyclooxygenase (COX) and lipoxygenase (LOX), which are involved in the production of inflammatory mediators like prostaglandins and leukotrienes [13,14,15]. Additionally, ferulic acid can suppress the expression of pro-inflammatory genes and cytokines, such as tumor necrosis factor-alpha (TNF-α) and interleukins, which play key roles in promoting inflammation [16,17]. The beneficial actions of this compound do not end here and include the protection of vascular endothelial cells [18,19], anti-fibrosis effect [20,21], anti-apoptotic effect [22,23], anti-platelet effect [24,25], and others [26,27]. In general, ferulic acid from dietary sources and oral administration has low bioavailability due to factors like limited absorption in the gastrointestinal tract and rapid metabolism in the liver [28,29]. However, certain formulations or delivery systems, such as lipid-based formulations or nanostructured carriers, can improve its bioavailability [30,31]. Additionally, co-administration with other compounds, such as piperine or quercetin, may further increase the bioavailability of ferulic acid [32,33]. On the other hand, ferulic acid is well absorbed by the skin through cutaneous administration [34,35], which makes it widely used in the cosmetics industry [36,37,38].

The importance of solubility in pharmaceutical applications cannot be overstated, as it serves as a critical determinant influencing the efficacy, safety, and overall success of drug development and delivery [39,40,41]. Solubility profoundly impacts various stages of the pharmaceutical lifecycle, from initial drug formulation to therapeutic administration [42,43,44]. The ability of a compound to dissolve in a solvent directly influences its bioavailability, governing the rate and extent of absorption into the bloodstream and, consequently, its therapeutic effectiveness [45,46]. Furthermore, solubility plays a pivotal role in the stability of drug formulations, affecting factors such as shelf life and storage conditions [47,48]. Challenges arising from poor solubility necessitate higher doses to achieve therapeutic levels, potentially increasing the risk of adverse effects [49,50,51]. Therefore, understanding, predicting, and manipulating the solubility of drug molecules are essential endeavors for pharmaceutical scientists [52,53,54,55,56,57]. Various techniques, including salt formation, co-solvent systems, and nanoparticle delivery systems, are employed to enhance solubility and optimize drug performance [58,59,60,61]. However, experimental efforts are not the only means aimed at the development of safer and more efficient medications. Accurate solubility modeling and predictions aid in the selection of promising drug candidates, formulation optimization, and the design of more effective drug delivery systems [39,62,63,64].

The “green chemistry” approach [65,66], representing a paradigm shift in chemical research and industry and aiming to design and implement processes that minimize environmental impact and maximize sustainability, has been extremely useful also in the realm of pharmaceuticals [67,68]. As stated, solvents play a crucial role in various chemical processes, yet traditional organic solvents often pose significant environmental and health hazards. Therefore, green chemistry emphasizes the development and utilization of alternative solvents with minimal environmental impact [69,70]. An important class of these new solvents, combining environmental safety with high solubilization efficiency, are deep eutectic solvents (DESs) [71,72]. Defined as mixtures of two or more components that form a eutectic mixture with a melting point significantly lower than that of either individual component [73,74], DESs offer several advantages over conventional solvents, including low toxicity, biodegradability, and tunable physicochemical characteristics [75,76,77]. These properties contribute to the widespread use of eutectic solvent systems, also in the pharmaceutical field [78,79,80,81,82,83,84,85]. It is worth noting that DESs, due to their unique properties, are utilized in the extraction of phenolic compounds, including phenolic acids [86], which suggests they may exhibit significant affinity for ferulic acid. The aim of this work is to expand the knowledge of the solubility of the compound in question in DESs and to interpret the obtained results based on molecular modeling supported by machine learning.

## 2. Results and Discussion

### 2.1. Solubility of Ferulic Acid in DESs and DES–Water Mixtures

In the experimental part of the study, deep eutectic solvents were applied to solubilize ferulic acid. While DESs are known for being very effective solubilizers of many compounds, the addition of water to these systems can improve solubility even further [82,87], which was the reason for also including the aqueous mixtures of DESs in the study. The solubility of various compounds in water is usually intensely lower than in the eutectic systems, and the addition of excess amounts of water to the DES systems results in a decrease in the solubility of the considered solute. In this concentration range water can be considered an anti-solvent for DESs. However, small amounts of added water tend to promote the solubility of such a solute. The origins of the observed phenomenon are complex and involve the formation of nanostructures of DES components and water molecules [88,89]. In particular, the stabilization nano-structure around choline chloride or other hydrogen bond acceptor (HBA) components of DESs play an important role [88]. These clusters are remarkably stable, even at modest dilutions. This complex behavior indicates that aqueous–DES mixtures cannot be regarded as typical antisolvent–cosolvent systems, although the favorable effect of the addition of water is still practically valuable and worth in-depth exploration. Here, an extensive search for the optimal water–DES solvent was performed while tuning ferulic acid solubility. In the first phase of experiments, the FA solubility was determined in pure DES systems. These DESs comprised either choline chloride (ChCl) or betaine (BI), both acting as hydrogen bond acceptors (HBA), and one of six polyols, namely ethylene glycol (ETG), diethylene glycol (DEG), triethylene glycol (TEG), 1,3-butanediol (B3D), 1,2-propanediol (P2D), and glycerol (GLY), acting as hydrogen bond donors (HBD). Three molar ratios of HBA to HBD were tested, i.e., 1:1, 1:2, and 1:4. All of the measurements at this screening phase were conducted at 25 °C.

The obtained results show that in general, DESs based on choline chloride are more efficient solubilizers of FA than those comprised of betaine. Nonetheless, the results present a similar overview, regardless of whether ChCl or BI were used as HBAs. When analyzing the molar compositions of the tested systems, it turns out that the 1:2 HBA–HBD molar ratio performs best, followed by the 1:4 ratio, with the 1:1 ratio being the least effective. This general observation is only altered in a few cases in the systems with betaine, where the 1:4 molar ratio outperforms some of the 1:2 systems. The comparison of the effectiveness of individual HBDs reveals differences between systems with choline chloride and betaine. When ChCl is used, the decreasing order of FA solubility is obtained, regardless of the molar ratio of DES constituents: TEG > DEG > GLY > ETG > B3D > P2D. Slightly more complex behavior is observed for systems utilizing betaine. For the optimal 1:2 molar composition, the following trend is obtained: TEG > GLY > DEG > ETG > B3D > P2D. Rather surprisingly, in the 1:4 molar composition, the DES prepared using B3D is the second most effective system, with the one using ETG being the least effective. Overall, it seems that three HBDs stand out as being the most promising, i.e., triethylene glycol, diethylene glycol, and glycerol. The ChCl-TEG eutectic system in a 1:2 molar proportion was the most effective DES among the studied formulations. At 25 °C, the solubility of ferulic acid was found to be x_FA_ = 0.0532. Using the ChCl-DEG system resulted in an FA solubility of x_FA_ = 0.0494, closely followed by the ChCl-GLY eutectic, characterized by x_FA_ = 0.0485. DES formulations with betaine were less efficient in terms of FA solubilization, and the top three systems, namely BI-TEG, BI-DEG, and BI-GLY, were responsible for ferulic acid solubilities of x_FA_ = 0.0432, x_FA_ = 0.0401, and x_FA_ = 0.0392 at 25 °C. Detailed results of FA solubility determination in the studied systems are presented in the Supplementary Materials (please refer to Table S1).

The obtained solubility values of FA in pure DESs served as a starting point for selecting the most promising eutectic systems, which would be used to form aqueous mixtures. Two systems utilizing choline chloride were selected along with two containing betaine, namely ChCl-TEG, ChCl-DEG, BI-TEG, and BI-GLY. The created ternary mixtures were characterized by different amounts of the DES, expressed as its mole fraction in a solute-free solution. The solubility of FA in such systems was measured at four temperatures in the range of 25 °C to 40 °C.

**Figure 1:**
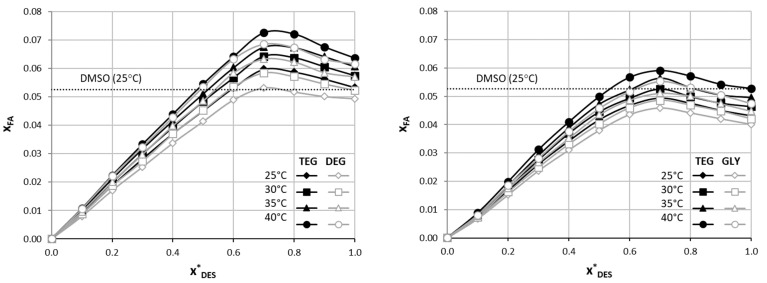
The solubility curves of ferulic acid (FA) in aqueous DES mixtures involving choline chloride (**left panel**) and betaine (**right panel**) and selected polyols at various temperatures, expressed as solvent-composition-related mole fractions. X*_DES_ stands for mole fractions of solute-free DES in aqueous mixtures. For comparison, the room-temperature solubility of ferulic acid in DMSO is provided.

As it was expected based on previous experiences, small amounts of water added to the eutectic solvent increase the solubility of ferulic acid. As stated, the nature of DES–water systems is complex. However, it is useful to describe their behavior at least as an apparent cosolvency/antisolvency effect. The solubility curves, describing the mole fraction solubility of FA as a function of the composition of ternary aqueous DES mixtures, are presented in Figure 1. The increasing amount of DES in its aqueous mixture leads to the increase of the solubility of ferulic acid, however, after a certain point, which represents the most effective composition, solubility starts to decrease. The composition characterizing the mixture yielding the highest FA solubility corresponds with the molar composition of x^*^_DES_ = 0.7 in the aqueous mixture. This observation holds for systems using both choline chloride and betaine at all studied temperatures. In the case of the mixture based on the ChCl-TEG eutectic, the solubility of ferulic acid at the optimal composition equaled x_FA_ = 0.0588 at 25 °C, which is 112% of the FA solubility in the pure eutectic solvent. For the second-most effective DES, namely ChCl-DEG, the FA solubility was found to be x_FA_ = 0.0532 at 25 °C, which corresponds to 108% of the FA solubility in the DES itself. For the aqueous mixtures of eutectics comprising betaine, the BI-TEG and BI-GLY systems were characterized with the solubility of ferulic acid equal x_FA_ = 0.0494 and x_FA_ = 0.0459, respectively, at 25 °C and at the optimal composition, which stands for about 114% of the water-free DES solubility. Additionally, to no surprise, the elevated temperature of the measurements resulted in increased solubility of ferulic acid. This increase was rather stable, and the difference between the solubility at 25 °C and at 40 °C amounted to around 20% regardless of the eutectic type and aqueous mixture composition. Detailed solubility values are again provided in Supplementary Materials (please refer to Tables S2 and S3).

The obtained results are worth comparing to solubility data available in the literature. The solubility of ferulic acid was studied in several systems, including water [90,91]; ethyl lactate and its mixtures with water [92]; isopropanol and its aqueous mixtures [93]; and a number of organic solvents, such as DMSO, trascutol, methanol, and ethyl acetate [91,94]. When considering these data, the following decreasing order of FA solubility (measured at 25 °C) in various neat solvents can be obtained: DMSO (x_FA_ = 0.0526) > transcutol (x_FA_ = 0.0430) > methanol (x_FA_ = 0.0295) > propylene glycol (x_FA_ = 0.026)3 > ethanol (x_FA_ = 0.0254) > ethylene glycol (x_FA_ = 0.0207) > isopropanol (x_FA_ = 0.0194) > 2-propanol (x_FA_ = 0.0188) > 2-butanol (x_FA_ = 0.0168) > 1-butanol (x_FA_ = 0.0161) > ethyl acetate (x_FA_ = 0.0130) > water (x_FA_ = 0.000049). In the above context, it turns out that among the neat DESs, only the ChCl-TEG 1:2 system offers a slightly better FA solubility than the most efficient classical solvent, namely DMSO, with the ChCl-DEG 1:2, ChCl-GLY 1:2, ChCl-ETG 1:2, and ChCl-TEG 1:4 systems being slightly less effective although outperforming the second classical solvent, which is transcutol. Among the systems with betaine, only the BI-TEG 1:2 system performed better than transcutol. The addition of water to the selected eutectics increased the FA solubility. Thus, the ChCl-TEG 1:2 (x*_DES_ = 0.7) and ChCl-DEG 1:2 (x*_DES_ = 0.7) systems gave higher solubility than DMSO, with the BI-TEG 1:2 (x*_DES_ = 0.7) being close to that.

### 2.2. Ferulic Acid Intermolecular Interactions in DES

**Figure 2:**
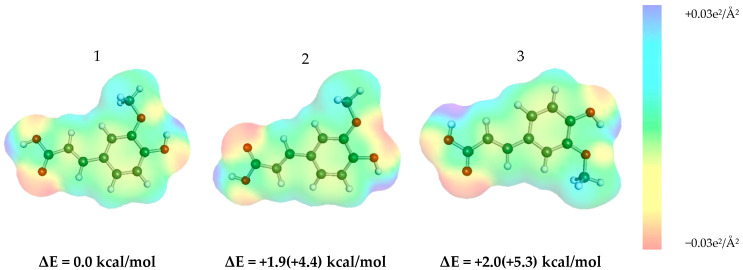
Schematic representation of ferulic acid conformers with electron density distributions and their relative energies in the bulk state, represented by an infinite conductor. Additionally, in parentheses are the relative values of total energies obtained for the gas phase.

The determination of the structure of ferulic acid in its monomeric form was the starting point of the computational procedure. Within a 5 kcal/mol energy window, three of the most stable and distinct conformers were identified, as schematically presented in Figure 2, where the relative energy values corresponding to the RI-BP97/def2-SVPD//RI-BP/def2-TZVPD-FINE level of theory were included. It is noteworthy that the most stable conformers differ in the orientation of the hydroxyl group connected to the aromatic ring or the hydroxyl group that is part of the carboxylic substituent. The most stable conformer forms an intramolecular hydrogen bond between the hydroxyl and methoxy groups. Through comparison with the second conformer, it can be inferred that this type of interaction contributes approximately 1.9 kcal/mol to intramolecular stabilization. The third conformer is similar to the first one but has an anti-rotated hydroxyl group in the carboxylic moiety, resulting in an energy increase of about 2.0 kcal/mol. The order of conformers remains consistent in both the bulk phase and vacuum.

**Figure 3:**
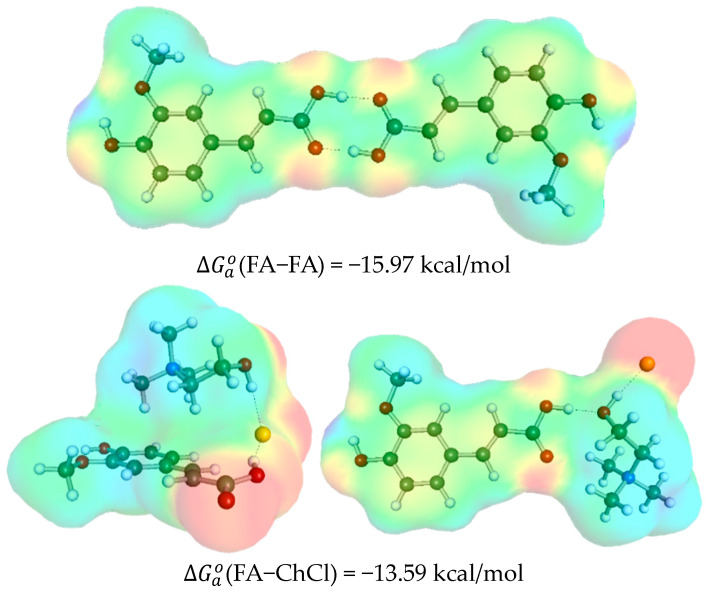

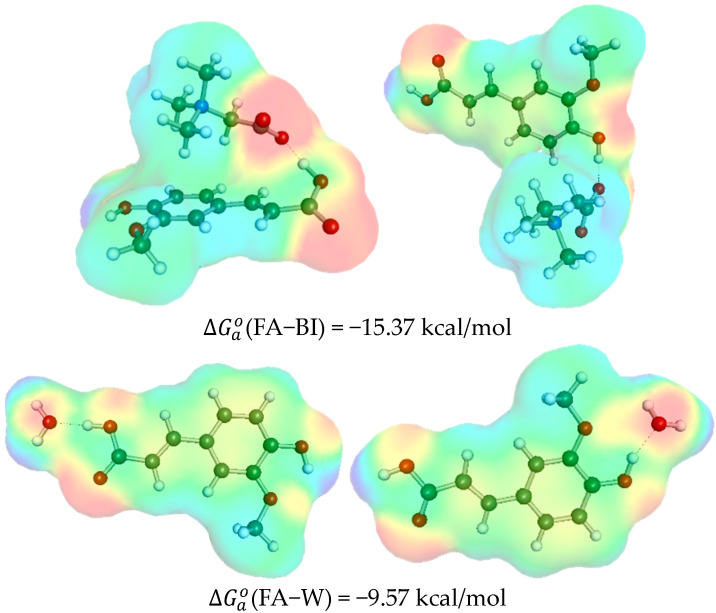
The most representative structures of FA dimers and hetero-molecular pairs formed with choline chloride, betaine, and water.

**Figure 4:**
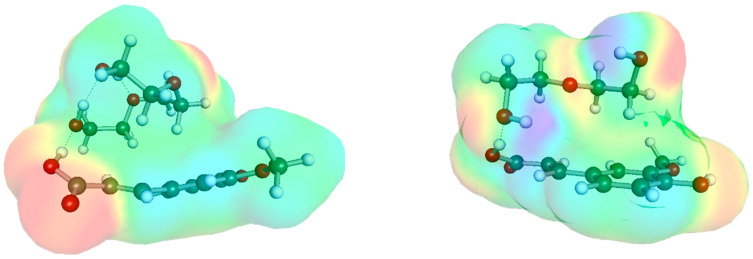

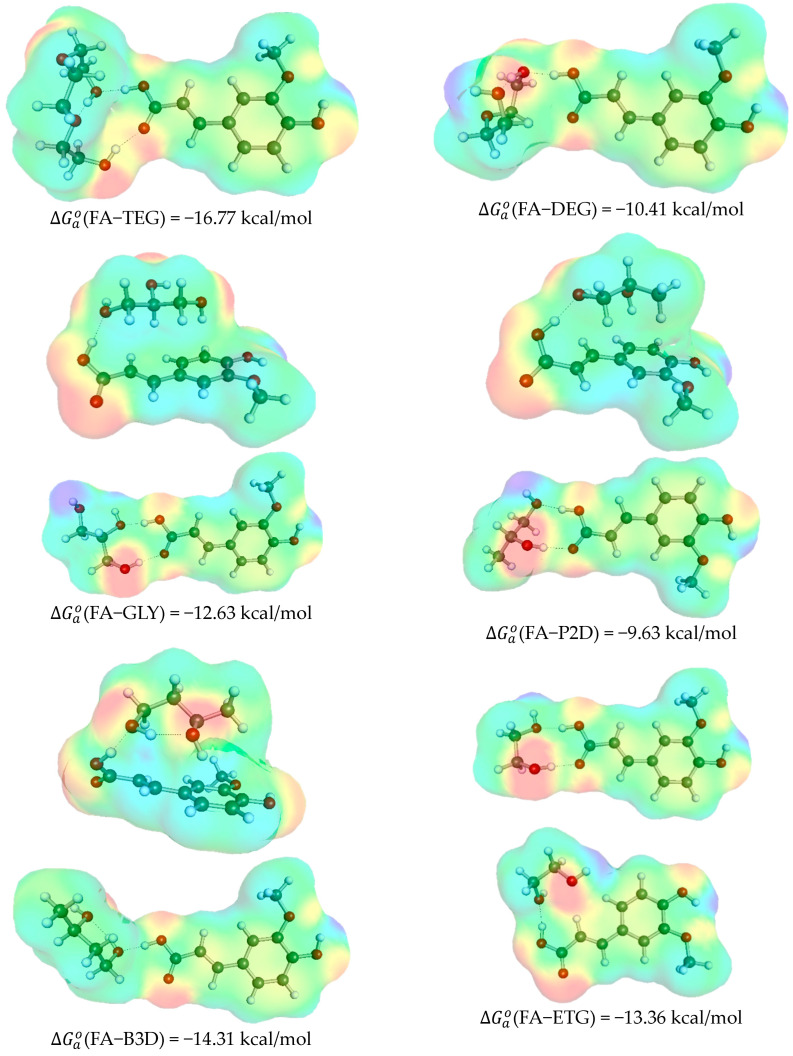
The most representative structures of FA with HBD counterparts of studied DESs.

The study of intermolecular interactions with DES constituents involved pairing each conformer of ferulic acid and minimizing the supermolecule energy. The most representative results are presented in Figure 3 and Figure 4. The affinities of ferulic acid in the studied DES were expressed using the concentration-independent standard Gibbs free energy values (${\Delta G}_{a}^{o}=RTln(a^{o})$) for the corresponding pair formation reactions at ambient conditions (T = 298.15 K). The subscript “a” indicates the use of mole fraction values corrected with activity coefficients, $a_{i}^{o}{=x}_{i}^{o}\cdot f_{i}^{o}$. This expression method is convenient, as it characterizes the thermodynamic propensity of interacting components regardless of the solvent environment. It is expected that the self-association behavior of ferulic acid is consistent across all systems, independent of the content and mole fractions of the components. Indeed, ferulic acid shows a strong propensity for self-aggregation, which can be inferred from the dimer structure and standard Gibbs free energy values, indicating the highest affinity among all studied pairs, as shown in Figure 3 and Figure 4. The FA dimer is stabilized by strong bidirectional hydrogen bonds forming a cyclic structure of the $C_{2}^{8}$ type, common for all carboxylic acids. Additionally, the carboxylic group in FA acts as a strong proton donor, directly contributing to the stabilization of both FA-ChCl and FA-BI pairs. Interestingly, two types of structural motifs were identified. The first type involves the third stable conformer of monomeric FA, where the energy disfavor of this conformer is compensated by non-polar interactions of ChCl or BI with the delocalized electrons of the aromatic ring, making these pairs the most stable. In the case of choline chloride, an alternative structure is stabilized by interactions with the hydrogen carboxylic group. Betaine, having a non-neutralized acetate group, cannot form stable pairs of this type and instead interacts with the hydroxyl group in the para position of FA. Notably, the affinity of FA to BI is significantly stronger compared to ChCl. Interactions between FA and water are much weaker and primarily involve hydrogen bonding with the carboxylic or hydroxyl groups. These observations suggest that the variety of potential contacts between FA and DES constituents might account for the high solvation abilities and consequently high solubility promoted by strong solute–solvent interactions.

Ferulic acid can also form stable pairs with all the hydrogen bond donor (HBD) counterparts of the DESs studied here. It is noteworthy that all polyols form two distinct motifs involving direct contact with the carboxylic group. In all cases, similarly to FA-ChCl interactions, the rotation of the hydroxyl group within the carboxylic moiety into the anti-position allows for additional interactions with the delocalized aromatic electron clouds. This behavior is observed for all polyols except ethylene glycol (ETG), which—due to its shortest chain—is unable to adopt a similar position to other proton-donating constituents. This prevents ETG from positioning above the aromatic ring of FA, making a structure stabilized by two hydrogen bonds the most probable configuration.

In all other cases, the non-polar interactions involving the aromatic electron cloud play a crucial role. These interactions not only offset the energy increase caused by the distortion of the carboxylic group but also contribute significantly to the overall stability of the structure. Notably, the highest affinity of FA was found for triethylene glycol (TEG), which correlates well with the highest solubility of FA in DESs containing this polyol regardless of the type of DBA. However, a linear trend between solubility and affinity values is not generally observed, suggesting that other factors contribute to the stabilization of the saturated FA-DES systems.

### 2.3. Machine Learning Model

Solubility measurements, though simple, are time-consuming experiments with several tricky steps, particularly when using DES as dissolution media. This complexity hinders the exhaustive search for new solvents, given the vast number of potential HBA-HBD combinations, along with concentration and temperature dependencies. Consequently, theoretical exploration of the solvent hyperspace to support experimental screening of the most suitable deep eutectic solvents including therapeutic variants (THEDES) is of significant practical value. In this study, a machine learning approach was employed, conducting an exhaustive search for models with the highest accuracy and predictive potential. A diverse set of non-linear regressors was tested for this purpose.

The training dataset comprised ferulic acid solubility values, including newly measured results for this study and previously published data. This dataset, the largest available at present, offers substantial structural diversity of solvents, which is a promising indicator for the generalization of the obtained model. The dataset (N = 344) includes mole fraction solubility values of FA in neat solvents (11 systems, N = 103), binary mixtures (1 system, N = 45), and DES (all presented in this paper, N = 196). The models were trained on a training subset (two-thirds of the entire set) by tuning the adjustable parameters of each regressor.

It is crucial to note that metrics such as mean absolute error (MAE) or the correlation coefficient (R^2^) were not the sole criteria for model accuracy. As mentioned in the methodology section, potential generalization was incorporated into the scoring function as a penalty derived from learning curve analysis (LCA) using scikit-learn. This approach evaluates model performance by increasing the percentage of included data and performing 10-fold cross-validation. This procedure, conducted separately for training and cross-validation subsets, provides comprehensive diagnostics by assessing the risk of overfitting and quantifying the models’ sensitivity to the used data. Although computationally expensive, this method ensures a thorough evaluation of the model.

**Figure 5:**
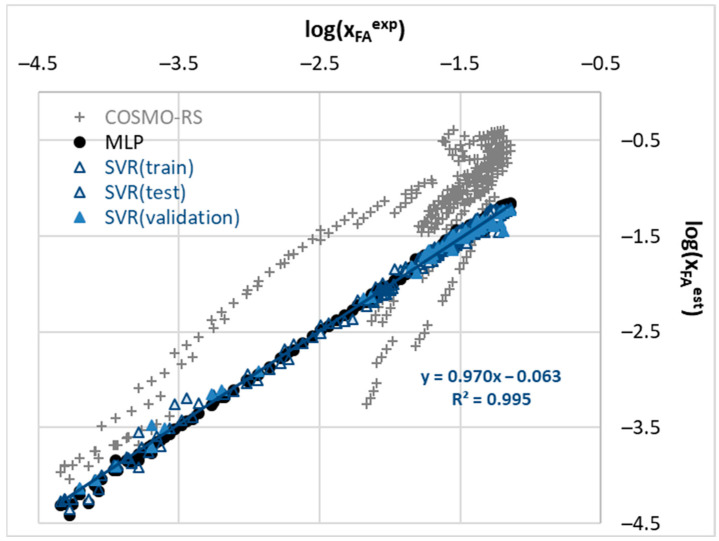
The correlation between experimental and computed solubility of ferulic acid in neat, binary, and deep eutectic solvents. The gray color denotes the results of COSMO-RS computations, blue indicates the SVR model, and in black, the results obtained from the MLP model are marked.

**Figure 6:**
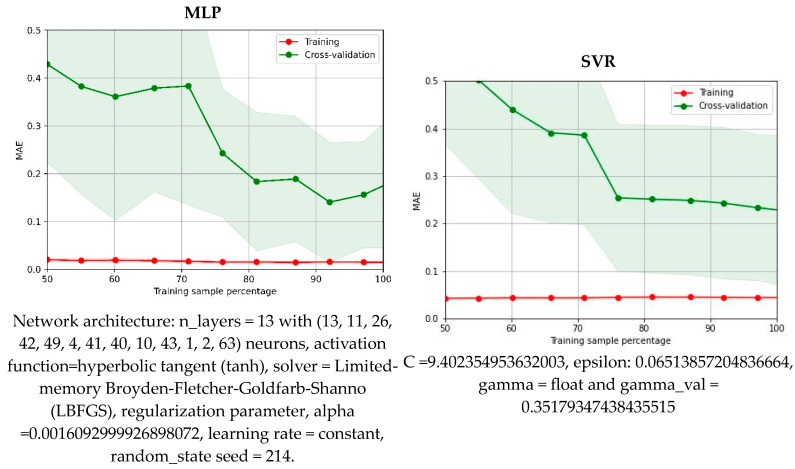

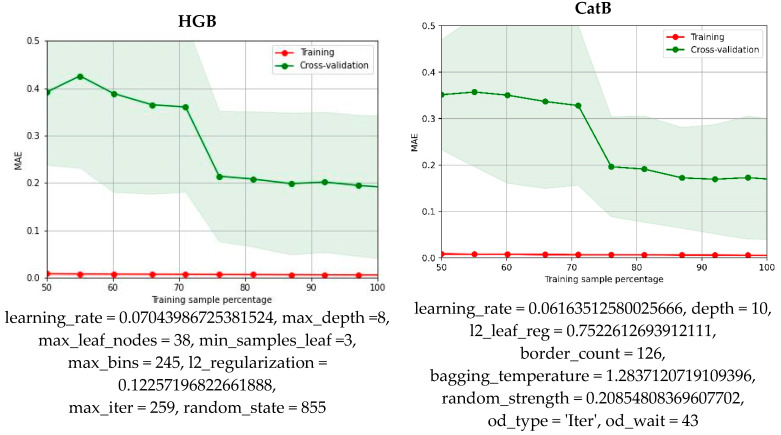
Characteristics of the best models found by hyper-parameters training for prediction of the ferulic acid solubility. The plots provide the results of the learning curve analysis, which is devoted to testing the consistency of models’ performance using both sub-sampling and cross-validation. The optimal values of each model are provided for reproducibility purposes.

Models characterized by low MAE, high R^2^, and failure to meet generalization criteria as revealed by LCA should be approached with caution. Indeed, this was observed in the best-performing model found in this study. As anticipated, utilizing artificial neural networks can lead to models capable of accurately back-computing experimental data. Multi-layer perceptrons (MLPs) were trained, allowing for flexible adjustment of network architecture to excel in capturing non-linear relationships. MLPRegressor, particularly adept at learning complex patterns through interconnected layers of neurons, demonstrated impressive accuracy. Figure 5 presents the accuracy of this regressor model, alongside other computations, including the second-best model, SVR, and solubility predictions based on the COSMO-RS approach. Two important conclusions can be drawn from this figure. Firstly, the native COSMO-RS results only qualitatively agree with experimental data, predicting general trends but not actual mole fraction solubility. The RMSD (root mean square deviation) and MAPE (mean absolute percentage error) for logarithmic mole fraction values are as high as 0.6 and 54.6%, respectively, which are significantly worse values compared to MLP and SVR, which had values of 0.025 (1.6%) and 0.061 (4.97%), respectively. Secondly, the predictions of both presented models are acceptable, though MLP outperforms SVR. However, the optimal architecture of the MLP network, optimized during the learning process, adopts a highly complex structure consisting of 13 hidden layers. The details of the optimized hyperparameters are provided in Figure 6.

It is noteworthy that the MLPRegressor and SVR were not the only non-linear models demonstrating acceptable accuracy in predicting ferulic acid solubility. Other less complex models, such as NuSVR, HistGradientBoosting, and CatBoost regressors, also achieved acceptable accuracy with significantly lower computational costs compared to MLP regressor. The best models can beranked as follows based on their MRSD and MAPE values given in the parenthesis, respectively: MLP (0.026, 1.57%) > SVR (0.062, 4.97%) ≈ NuSVR (0.063, 5.15%) > HGB (0.050, 2.16%) > CatBoost (0.051, 2.03%).

The optimized parameters for these models are summarized in Figure 6, which also includes the results of learning curve analysis for each regressor. It is noteworthy that considering multiple regressor models for experimental data modeling and prediction offers several advantages. Firstly, it can enhance performance by leveraging the strengths of different models, such as their ability to capture linear or non-linear relationships. Secondly, comparing and validating multiple models on the same dataset provides insights into their relative strengths, weaknesses, and overall reliability. Lastly, regressors can be used to formulate ensemble methods with optimized weights, further improving overall performance and generalization by mitigating bias and variance. This approach also promotes better generalization to new data and helps identify uncertainties or inconsistencies in predictions. However, this step was not implemented here, as the accuracy was acceptable even with a single-regressor approach and the available data pool was rather limited. For further examination of model properties, the LCA results are presented in Figure 6. Since NuSVR closely mirrors SVR results in both solubility data back computation and LCA results, it was excluded from further analysis. As previously noted, the MLP model has a complex structure that allows for the most accurate solubility back computations. However, LCA reveals significant limitations when applying this model to new data, as the learning curve shows considerable sensitivity to the data pool used for MLP application. Ideally, lines resulting from LCA should exhibit a smooth decrease in MAE with increasing data sample percentage for both training and cross-validation subsets. In the case of the MLP model, this requirement is met for the training dataset, suggesting excellent fitting to known experimental data, but values not seen in the learning phase are predicted with much less consistency. This suggests that while the complex architecture effectively captures the diversity of known data, it fails with new data, indicating poor generalizability for theoretical screening of FA solubility in new dissolution media. The second model analyzed in Figure 6 suffers much less from this drawback. Indeed, after the inclusion of at least 75% of the solubility data, a systematic decrease in MAE values is observed. Similar conclusions can be drawn for the other two models included in Figure 6. From a practical point of view, the selection of SVR, NuSVR, or HGB for further application is a fortunate circumstance since these models are relatively fast to optimize and to apply for screening purposes. Because of this, they are recommended for further development and wrapping with a user-friendly application. In general, both support vector regression and its variant, NuSVR, are very effective when dealing with high-dimensional data and can handle non-linear relationships through the use of kernel functions. Support vector regression aims to find a hyperplane that best fits the data while maximizing the margin. It uses a subset of training samples called support vectors to define the regression function. SVR is known for its ability to handle non-linear relationships by applying kernel functions, such as radial basis function (RBF), polynomial, or sigmoid. NuSVR utilizes additional parameters to control the number of support vectors. The HistGradientBoosting regressor belongs to the boosting family of algorithms and is characterized by an ensemble of decision trees. It utilizes a gradient boosting framework, where subsequent trees are built to correct the errors made by previous trees. It incorporates histogram-based gradient boosting, which improves training speed and memory efficiency. The CatBoost regressor, on the other hand, is a gradient boosting algorithm that is particularly effective with categorical features. It requires minimal data preprocessing and is known for its fast training speed and robust handling of various data types.

## 3. Materials and Methods

### 3.1. Materials

*Trans*-ferulic acid (FA, CAS: 537-98-4, MW = 194.18 g/mol) was supplied by Sigma Aldrich (St. Louis, MO, USA) with a purity of ≥99%. Two hydrogen bond acceptors were used in the formation of deep eutectic solvents, namely choline chloride (ChCl, CAS: 67-48-1) and betaine (BI, CAS: 107-43-7). Six different hydrogen bond donors were also used, i.e., ethylene glycol (ETG, CAS: 107-21-1), diethylene glycol (DEG, CAS: 111-46-6), triethylene glycol (TEG, CAS: 112-27-6), glycerol (GLY, CAS: 56-81-5), 1,2-propanediol (P2D, CAS: 57-55-6), and 1,3-butanediol (B3D, CAS: 107-88-0). The constituents of the eutectic systems were all delivered by Sigma Aldrich (St. Louis, MO, USA), and their purity was ≥99%. Methanol (CAS: 67-56-1), which was utilized as a secondary solvent throughout the studies, was obtained from Avantor Performance Materials (Gliwice, Poland) and had a purity of ≥99%. Choline chloride was dried before use, while all the other chemicals were used without any initial procedures.

### 3.2. Preparation of the Calibration Curve

The spectrophotometric determination of the solubility of ferulic acid in deep eutectic solvents and their aqueous mixtures was preceded by the preparation of a calibration curve. For this purpose, the stock solution of FA was prepared in a 100 mL volumetric flask using methanol as a solvent. This solution was then diluted, which was achieved by transferring fixed amounts of the stock solution into 10 mL volumetric flasks and adding methanol accordingly. Eleven solutions were obtained in this way, with varying concentrations in the range of 0.00618 mg/mL to 0.0206 mg/mL. The absorption spectra of these solutions were then recorded with the help of an A360 spectrophotometer from AOE Instruments (Shanghai, China) in the wavelength range from 200 nm to 500 nm. The absorbance maximum was found to correspond to the 321 nm wavelength and did not change over the course of the measurements. Three separate curves were prepared in this manner, and the final curve was the result of their averaging. The obtained linear regression was found to be A = 98.226 × C + 0.002 (A—absorbance, C—concentration in mg/mL). The validation parameters of the curve included the determination coefficient R^2^, the limit of detection (LOD), and the limit of quantification (LOQ). The R^2^ coefficient was equal to 0.9989, which ensures satisfactory linearity of the curve. LOD was found to be 0.000494 mg/mL, while LOQ was 0.001483 mg/mL, which are values far below the concentrations achieved in the studied samples. Overall, the calibration curve can be considered adequate for the determination of the solubility of ferulic acid.

### 3.3. Preparation of the Samples and Solubility Measurements

The solubility of ferulic acid in the considered eutectic systems was determined using the well-established and reliable shake-flask method [95,96,97,98] combined with spectrophotometric measurements.

Deep eutectic solvents were formed by combining a hydrogen bond acceptor with a hydrogen bond donor in various molar ratios, including a unimolar proportion, a 2-fold excess amount of the HBD, and its 4-fold excess amount. One of two substances was used as a HBA, i.e., choline chloride or betaine, while one of six polyols—i.e., TEG, DED, ETG, GLY, P2D, or B3D—was used as an HBD. This resulted in a total of 36 eutectic systems. In order to prepare the eutectic formulation, the two constituents were mixed in glass vessels in a specific molar ratio, placed on a heating plate, and further mixed until the formation of a homogenous solution. DESs prepared in this manner were used in their pure form, and the most promising ones were also selected to form mixtures with water in pre-determined molar compositions.

As the initial step in the solubility determination procedure, saturated solutions of ferulic acid in considered DESs were prepared. This was done by placing an excess amount of FA in a test tube and adding the selected DES or aqueous DES mixture. The Orbital Shaker Incubator ES-20/60 from Biosan (Riga, Latvia) was used to ensure a stable temperature of 25 °C, 30 °C, 35 °C, or 40 °C, depending on the measurement conditions, for 24 h of incubation with simultaneous mixing at 60 rev/min. The samples were then filtered through a 0.22 µm pore-size PTFE syringe filter. All of the test tubes, syringes, pipette tips, and filters were initially heated at the same temperature as the measured sample in order to prevent precipitation. The samples were accordingly diluted with methanol before the measurements. As was the case for the calibration curve, the A360 spectrophotometer was used to record the spectra of the samples in the 200 nm–500 nm wavelength range with a 1 nm resolution and using methanol for calibration. The concentration of ferulic acid was calculated based on the linear equation of the calibration curve and the absorbance values recorded at the characteristic wavelength, i.e., 321 nm. Additionally, the density of the samples was measured, which was necessary for the computation of mole fraction solubility. For this propose, 1 mL of each solution was weighed in a 10 mL volumetric flask using a RADWAG (Radom, Poland) AS 110 R2.PLUS analytical balance with 0.1 mg precision. For each studied system, three samples were prepared and measured, and the obtained values were averaged.

### 3.4. Conformational Analysis

The most representative structures of either monomeric forms of every DES constituent or their homo- and hetero-molecular pairs were found by employing an extensive conformational analysis using the COSMOconf [99] and COMSOtherm [100] packages. The procedure was already described in our previous papers [101,102,103], hence only a brief synopsis is provided here. Each molecule, or molecular complex, was represented by a maximum of ten low-energy conformations identified by independent conformational searches for both the gas and condensed phases. The latter is crucial to account for the influence of the surrounding environment within the conductor-like screening model. The outcome of this protocol is a set of “cosmo” and “energy” files compatible with the latest parameter set, which is BP_TZVPD_FINE_24.ctd. This file comprises all the necessary parameters utilized for thermodynamic properties’ computation in COMSOtherm [100]. It is worth mentioning that there are available variants of “ctd” files comprising parameter values adjusted for different levels of computation. Here, the RI-BP/def2-TZVPD-FINE level was used as the final step of the conformational analysis and affinity computations. In the manual, there is a recommended two-step procedure starting with geometry optimization on a slightly less demanding level, namely RI-BP/def-TZVP, followed by the final single-point energy computations and generation of the necessary “cosmo” and “energy” files. This procedure can be followed for many molecules but suffers from serious drawbacks if applied to intermolecular complexes or flexible molecules stabilized by non-bonding type of interactions. It is related to inadequate representation of the dispersive forces by the RI-BP/def-TZVP method, and adding correction to the final energy cannot correct the occasionally obtained improper geometries. This is especially important for complexes with delocalized electrons, the interactions of which are poorly represented using the RI-BP/def-TZVP approach. For example, many stacking complexes are not predicted to be stable. This problem was already stated for edaravone [101] or methylxhanthines [97,104]. Hence, the actual geometry optimization was performed for both monomers and pairs using the RI-BP97/def2-SVPD approach. Apart from the more realistic pair geometries, there is an additional benefit of using this level of computations, namely the straightforward accounting for basis superposition set error (BSSE). This is regarded as an important component of the total interaction energy. However, rigorous computations of the BSSE via counterpoise correction can be time-consuming. Therefore, much less demanding alternatives using the DFT-C approach were proposed [105]. This geometry-based method of BSSE estimation relies on BP97/def2-SVPD geometries and accounts for atom–atom many-body corrections to the total molecular complex energy. Hence, this method was used for the geometry optimization of all structures used in this study. The molecular geometries obtained in such a manner were then used for single energy computations at a level compatible with the parametrization of COSMOtherm mentioned above. Hence, the full acronym describing this approach is as follows: RI-BP97/def2-SVPD//RI-BP/def2-TZVPD-FINE, where the first section before the double slash represents the optimization level and the second part the single energy computations.

The conformational search of the most probable structures or monomers is straightforward and done automatically using COSMOconf. However, identification of the most representative pair conformations posed a more significant computational challenge. Here, the same protocol as previously used for other APIs [101,102,103] was used. This involved generating conformations for various combinations of ferulic acid dimers and ferulic acid-solvent pairs. To achieve this, the COSMOtherm software employed the “CONTACT = {1 2} ssc_probability ssc_weak ssc_ang = 15.0” command. This command prioritizes the generation of the most probable pairs based on contact probabilities considering both hydrogen bonding and weak interactions. Consequently, this step typically results in a substantial number of initial structures requiring further optimization according to the above procedure with the implementation of data reduction to eliminate redundant and high-energy geometries. Two criteria were used for this selection: root-mean-square deviation (RMSD) and relative energy compared to the most stable conformer. Ultimately, only unique contacts within a 5.0 kcal/mol energy window relative to the most stable structure were retained for each complex, ensuring a representative pool of conformers with diverse structures and energies. The final “cosmo” and “energy” files were generated on the same level of theory and used for affinity computations using COSMOtherm parametrization. The core of all quantum chemistry computations was the Biovia TURBOMOLE [106] interfaced with TmolX. In the final step, the affinity computations were performed to characterize the interaction properties of ferulic acid with every constituent of the studied DESs using the default settings of COSMOtherm.

### 3.5. Solubility Dataset

The solubility dataset was defined based on both literature studies and augmented with new measurements presented in this paper. The literature review led to finding the data on FA solubility in the following neat solvents, methanol [91,94], ethanol [91,94,107], isopropanol [91,93,94], 1-butanol [91], 2-butanol [91], ethylene glycol [91], propylene glycol [91], transcutol [91], DMSO [91], ethyl acetate [91,92,94], and water [90,91,93,94,107]. Additionally, there are also available data for aqueous mixtures of isopropanol [93]. It happened that the highest solubility was found to be in DMSO.

### 3.6. Machine Learning Protocol

The machine learning protocol utilized in this study adheres to the methodology previously applied in our earlier projects [79,102,108]. As comprehensive details have been previously reported, only brief remarks are provided here. The solubility prediction model was developed using in-house Python code (version 3.10, https://www.python.org/) designed for hyperparameter tuning across 36 regression models. These models encompass a wide range of algorithms, including linear models, boosting methods, ensembles, nearest neighbors, neural networks, and other regressor types. Hyperparameter optimization was conducted using Optuna (version 3.2, https://optuna.org/), an open-source Python package. The optimization process involved 5000 minimization trials, employing the tree-structured Parzen estimator (TPE) as the search algorithm sampler. To evaluate the performance of each regression model, a custom scoring function was defined, integrating multiple metrics to assess both accuracy and generalizability, as detailed in a previous work [108]. This scoring function includes penalties derived from learning curve analysis (LCA), performed using the scikit-learn library (version 1.2.2) during the parameter tuning process. Due to the computational demands of LCA, initial computations were limited to two points, encompassing 50% and 100% of the total dataset. Subsequent LCA evaluations of the final model involved 20-point calculations within the 50–100% data range. The custom loss function incorporates the mean MAE values obtained from the largest training set size, thereby integrating both accuracy and generalizability aspects and providing insights into the model’s performance on unseen data.

### 3.7. Molecular Descriptors

The set of molecular descriptors was formulated as described in our previous publication [103], based on the σ-potential values. The temperature-dependent σ-potentials were calculated for each compound in its pure, single-component state. The molecular descriptors for complex systems, utilized in machine learning, were represented as the difference between the σ-potential of a pure solute and that of a solvent at a given temperature. For multicomponent solvents, the σ-potential was characterized as the pure state value weighted by the mole fraction of the solute-free mole fraction. Typically, COSMOtherm generates σ-potential profiles consisting of 61 points for σ values between −0.03 and +0.03 e/Å^2^ with a 0.001-step increment.

**Figure 7:**
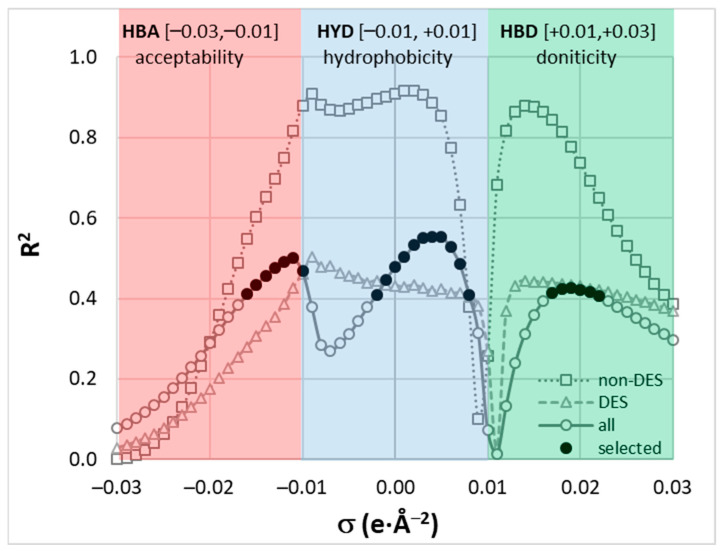
The correlation between relative solute-solvent σ-potentials and experimental solubility expressed as a function of σ values. Two series represent correlations computed for subsets including only non-DES solvents (neat solvents and binary mixtures) or only DES systems. Bold symbols define regions used as a set of molecular descriptors, where R^2^ > 0.4 for either subset. The split into three distinct subranges is marked with colorful rectangles.

To reduce the number of descriptors, the most promising subset was identified by inspecting the significance of the relationship between experimental solubility and relative σ-potential values. This selection criterion was quantified by restriction to R^2^ > 0.4 for a given σ value. This is illustrated in Figure 7, which shows two subsets: non-DES solvents (including neat solvents and binary mixtures) and DES systems. Notably, the overall correlation between experimental solubility and σ-potential is modest when considering the entire dataset. However, restricting the analysis to non-DES systems reveals a very high correlation (R^2^ > 0.8) in the non-polar region, typically attributed to hydrophobicity (HYD). Additionally, the sub-range of hydrogen bond donicity (HBD) shows a high correlation with FA solubility. The points marked with bold black dots in Figure 7 were used for machine learning.

Additionally, solubility was computed by fully solving the solid-liquid equilibrium (SLE) problem using COSMOtherm [100] for two reasons. Firstly, it is interesting how accurate are the predictions based on the COSMO-RS theory [109]. Secondly, the computed solubility can be used as a molecular descriptor for machine learning purposes. It is crucial to note that the COSMO-RS approach is designed for predicting the thermodynamic characteristics of bulk systems, excluding the solid state. Since solubility involves the transition of the crystalline phase into a liquid saturated solution, the contribution of fusion data to the overall thermodynamic characteristics must be added to the input files. The required data include the melting temperature (T_m_ = 445.83 K), heat of fusion (ΔH_fus_ = 32.49 kJ/mol), and heat capacity change upon melting (ΔC_p,fus_ ≈ ΔS_fus_ ≈ ΔH_fus_T_m_). The values in parentheses correspond to the average data provided in the compilation by Acree et al. [110]. The obtained solubility values were utilized as molecular descriptors in addition to the relative σ-potential values previously defined.

## 4. Conclusions

Ferulic acid is an important representative of phenolic acids with many practical applications. Since it can be obtained from natural sources, the selection of the most effective and green media for extraction seems to be of immense importance. This is emphasized by efforts to measure the solubility of FA in neat solvents and binary mixtures, as documented by a study of the literature. This paper further extends the knowledge of the dissolution of ferulic acid through a systematic study of two types of deep eutectic solvents involving choline chloride or betaine, acting as hydrogen bond acceptors, with one of six polyols playing the role of hydrogen bond donors. The performed optimization encompassed both the DES composition and its relative concentration in aqueous mixtures. It was found that the eutectics utilizing choline chloride were slightly more effective than those with betaine and that a 1:2 molar ratio of the HBA and HBD counterparts of the eutectic was the optimal one. Furthermore, the addition of small amounts of water to the DES further promotes the solubility of FA compared to the neat eutectic. Among the considered polyols, triethylene glycol proved to be the most effective. The presented results suggest that designed solvents comprising choline chloride and TEG, with an addition of water, can be treated as efficient alternatives to traditional organic solvents, including the first-choice solvent, namely DMSO. In order to gain insight into the saturated systems of FA in liquid media on a molecular level, the intermolecular interactions in the considered systems were studied, which led to the identification of the most stable homo- and hetero-molecular pairs formed between the interacting compounds. For the purpose of solvent screening, aimed at assisting the time-consuming experimental phase, a machine learning protocol was employed for the formulation of a non-linear model being able to predict FA solubility. This phase resulted in obtaining very accurate models that were able to precisely back-compute solubility as a function of solvent type and temperature. It is recommended to utilize the SVR regressor with the provided values of optimized parameters for screening purposes of new dissolution media. It is also worth mentioning that the very popular way of solubility prediction using the COMSO-RS approach is at most qualitatively accurate, and the developed models are much more accurate.

## Data Availability

All data supporting the reported results are available on request from the corresponding author.

## Supplementary Materials

The following supporting information can be downloaded at: https://www.mdpi.com/article/10.3390/molecules29163841/s1, Table S1: Mole fraction solubility of ferulic acid (IP) in neat DES containing either choline chloride or betaine as a HBA and one of six studied polyols as a HBD in various molar proportions at 25 °C. Standard deviation values are given in parentheses; Table S2: Mole fraction solubility of ferulic acid (FA) in aqueous-choline-chloride-based DES mixtures at various temperatures. In the first column, x*DES denotes the mole fraction of DES in solute-free mixtures with water. Standard deviation values are given in parentheses; Table S3: Mole fraction solubility of ferulic acid (FA) in aqueous-betaine-based DES mixtures at various temperatures. In the first column, x*DES denotes the mole fraction of DES in solute-free mixtures with water. Standard deviation values are given in parentheses.

## References

[1] Ou S., Kwok K.C. (2004). Ferulic acid: Pharmaceutical functions, preparation and applications in foods. J. Sci. Food Agric..

[2] Mancuso C., Santangelo R. (2014). Ferulic acid: Pharmacological and toxicological aspects. Food Chem. Toxicol..

[3] Li D., Rui Y.X., Guo S., Luan F., Liu R., Zeng N. (2021). Ferulic acid: A review of its pharmacology, pharmacokinetics and derivatives. Life Sci..

[4] Babbar R., Dhiman S., Grover R., Kaur A., Arora S. (2021). A Comprehensive Review on Therapeutic Applications of Ferulic Acid and its Novel Analogues: A Brief Literature. Mini-Rev. Med. Chem..

[5] Kumar N., Pruthi V. (2014). Potential applications of ferulic acid from natural sources. Biotechnol. Rep..

[6] Sakai S., Kawamata H., Kogure T., Mantani N., Terasawa K., Umatake M., Ochiai H. (1999). Inhibitory Effect of Ferulic Acid and Isoferulic Acid on the Production of Macrophage Inflammatory Protein-2 in Response to Respiratory Syncytial Virus Infection in RAW264.7 Cells. Mediat. Inflamm..

[7] Graf E. (1992). Antioxidant potential of ferulic acid. Free Radic. Biol. Med..

[8] Zduńska K., Dana A., Kolodziejczak A., Rotsztejn H. (2018). Antioxidant Properties of Ferulic Acid and Its Possible Application. Ski. Pharmacol. Physiol..

[9] Kikuzaki H., Hisamoto M., Hirose K., Akiyama K., Taniguchi H. (2002). Antioxidant properties of ferulic acid and its related compounds. J. Agric. Food Chem..

[10] Urbaniak A., Szelag M., Molski M. (2013). Theoretical investigation of stereochemistry and solvent influence on antioxidant activity of ferulic acid. Comput. Theor. Chem..

[11] Borges F., Lima J.L.F.C., Pinto I., Reis S., Siquet C. (2003). Application of a Potentiometric System with Data-Analysis Computer Programs to the Quantification of Metal-Chelating Activity of Two Natural Antioxidants: Caffeic Acid and Ferulic Acid. Helv. Chim. Acta.

[12] Truong D.H., Nhung N.T.A., Dao D.Q. (2020). Iron ions chelation-based antioxidant potential vs. pro-oxidant risk of ferulic acid: A DFT study in aqueous phase. Comput. Theor. Chem..

[13] Hosoda A., Ozaki Y., Kashiwada A., Mutoh M., Wakabayashi K., Mizuno K., Nomura E., Taniguchi H. (2002). Syntheses of Ferulic Acid Derivatives and Their Suppressive Effects on Cyclooxygenase-2 Promoter Activity. Bioorg. Med. Chem..

[14] Kumar N., Pruthi V. (2015). Structural elucidation and molecular docking of ferulic acid from Parthenium hysterophorus possessing COX-2 inhibition activity. 3 Biotech.

[15] Amić A., Marković J.M.D., Marković Z., Milenković D., Milanović Ž., Antonijević M., Cagardová D.M., Pedregal J.R.G. (2021). Theoretical study of radical inactivation, lox inhibition, and iron chelation: The role of ferulic acid in skin protection against uva induced oxidative stress. Antioxidants.

[16] Zhao J., Suyama A., Tanaka M., Matsui T. (2014). Ferulic acid enhances the vasorelaxant effect of epigallocatechin gallate in tumor necrosis factor-alpha-induced inflammatory rat aorta. J. Nutr. Biochem..

[17] Ganesan R., Rasool M. (2019). Ferulic acid inhibits interleukin 17-dependent expression of nodal pathogenic mediators in fibroblast-like synoviocytes of rheumatoid arthritis. J. Cell. Biochem..

[18] Wang J., Yuan Z., Zhao H., Ju D., Chen Y., Chen X., Zhang J. (2011). Ferulic acid promotes endothelial cells proliferation through up-regulating cyclin D1 and VEGF. J. Ethnopharmacol..

[19] Serreli G., Le Sayec M., Thou E., Lacour C., Diotallevi C., Dhunna M.A., Deiana M., Spencer J.P.E., Corona G. (2021). Ferulic Acid Derivatives and Avenanthramides Modulate Endothelial Function through Maintenance of Nitric Oxide Balance in HUVEC Cells. Nutrients.

[20] Ali S.A., Saifi M.A., Pulivendala G., Godugu C., Talla V. (2021). Ferulic acid ameliorates the progression of pulmonary fibrosis via inhibition of TGF-β/smad signalling. Food Chem. Toxicol..

[21] Zhao X.M., Zhang J., Liang Y.N., Niu Y.C. (2020). Astragaloside IV Synergizes with Ferulic Acid to Alleviate Hepatic Fibrosis in Bile Duct-Ligated Cirrhotic Rats. Dig. Dis. Sci..

[22] Gupta A., Singh A.K., Loka M., Pandey A.K., Bishayee A. (2021). Ferulic acid-mediated modulation of apoptotic signaling pathways in cancer. Adv. Protein Chem. Struct. Biol..

[23] Nakayama H., Nakahara M., Matsugi E., Soda M., Hattori T., Hara K., Usami A., Kusumoto C., Higashiyama S., Kitaichi K. (2020). Protective Effect of Ferulic Acid against Hydrogen Peroxide Induced Apoptosis in PC12 Cells. Molecules.

[24] Zhang K., Shen X., Yang L., Chen Q., Wang N., Li Y., Song P., Jiang M., Bai G., Yang P. (2022). Exploring the Q-markers of *Angelica sinensis* (Oliv.) Diels of anti-platelet aggregation activity based on spectrum–effect relationships. Biomed. Chromatogr..

[25] Nguyen T.M.H., Le H.L., Ha T.T., Bui B.H., Le N.T., Nguyen V.H., Nguyen T.V.A. (2020). Inhibitory effect on human platelet aggregation and coagulation and antioxidant activity of *C. edulis* Ker Gawl rhizome and its secondary metabolites. J. Ethnopharmacol..

[26] Wang N.Y., Li J.N., Liu W.L., Huang Q., Li W.X., Tan Y.H., Liu F., Song Z.H., Wang M.Y., Xie N. (2021). Ferulic Acid Ameliorates Alzheimer’s Disease-like Pathology and Repairs Cognitive Decline by Preventing Capillary Hypofunction in APP/PS1 Mice. Neurotherapeutics.

[27] Dong X., Huang R. (2022). Ferulic acid: An extraordinarily neuroprotective phenolic acid with anti-depressive properties. Phytomedicine.

[28] Bourne L.C., Rice-Evans C. (1998). Bioavailability of Ferulic Acid. Biochem. Biophys. Res. Commun..

[29] Lafay S., Gil-Izquierdo A. (2008). Bioavailability of phenolic acids. Phytochem. Rev..

[30] Zhang Y., Li Z., Zhang K., Yang G., Wang Z., Zhao J., Hu R., Feng N. (2016). Ethyl oleate-containing nanostructured lipid carriers improve oral bioavailability of trans-ferulic acid ascompared with conventional solid lipid nanoparticles. Int. J. Pharm..

[31] Hassanzadeh P., Arbabi E., Atyabi F., Dinarvand R. (2018). Ferulic acid-loaded nanostructured lipid carriers: A promising nanoformulation against the ischemic neural injuries. Life Sci..

[32] Hithamani G., Srinivasan K. (2017). Bioavailability of finger millet (*Eleusine coracana*) phenolic compounds in rat as influenced by co-administered piperine. Food Biosci..

[33] Scholz S., Williamson G. (2013). Interactions Affecting the Bioavailability of Dietary Polyphenols in Vivo. Int. J. Vitam. Nutr. Res..

[34] Zhang L.W., Al-Suwayeh S.A., Hsieh P.W., Fang J.Y. (2010). A comparison of skin delivery of ferulic acid and its derivatives: Evaluation of their efficacy and safety. Int. J. Pharm..

[35] Chen M., Liu X., Fahr A. (2010). Skin delivery of ferulic acid from different vesicular systems. J. Biomed. Nanotechnol..

[36] Zduńska-Pęciak K., Dębowska R., Kołodziejczak A., Rotsztejn H. (2022). Ferulic acid—A novel topical agent in reducing signs of photoaging. Dermatol. Ther..

[37] Janus E., Pinheiro L.R., Nowak A., Kucharska E., Świątek E., Podolak N., Perużyńska M., Piotrowska K., Duchnik W., Kucharski Ł. (2023). New Ferulic Acid and Amino Acid Derivatives with Increased Cosmeceutical and Pharmaceutical Potential. Pharmaceutics.

[38] Cavalcanti G.R., Duarte F.I.C., Converti A., de Lima Á.A.N. (2020). Ferulic Acid Activity in Topical Formulations: Technological and Scientific Prospecting. Curr. Pharm. Des..

[39] Martínez F., Jouyban A., Acree W.E. (2017). Pharmaceuticals solubility is still nowadays widely studied everywhere. Pharm. Sci..

[40] Savjani K.T., Gajjar A.K., Savjani J.K. (2012). Drug Solubility: Importance and Enhancement Techniques. ISRN Pharm..

[41] Coltescu A.R., Butnariu M., Sarac I. (2020). The importance of solubility for new drug molecules. Biomed. Pharmacol. J..

[42] Yang Z., Yang Y., Xia M., Dai W., Zhu B., Mei X. (2022). Improving the dissolution behaviors and bioavailability of abiraterone acetate via multicomponent crystal forms. Int. J. Pharm..

[43] Kalam M.A., Alshamsan A., Alkholief M., Alsarra I.A., Ali R., Haq N., Anwer M.K., Shakeel F. (2020). Solubility Measurement and Various Solubility Parameters of Glipizide in Different Neat Solvents. ACS Omega.

[44] Kim H.-S., Kim C.-M., Jo A.-N., Kim J.-E. (2022). Studies on Preformulation and Formulation of JIN-001 Liquisolid Tablet with Enhanced Solubility. Pharmaceuticals.

[45] Khadka P., Ro J., Kim H., Kim I., Kim J.T., Kim H., Cho J.M., Yun G., Lee J. (2014). Pharmaceutical particle technologies: An approach to improve drug solubility, dissolution and bioavailability. Asian J. Pharm. Sci..

[46] Müller C.E. (2009). Prodrug Approaches for Enhancing the Bioavailability of Drugs with Low Solubility. Chem. Biodivers..

[47] Das T., Mehta C.H., Nayak U.Y. (2020). Multiple approaches for achieving drug solubility: An in silico perspective. Drug Discov. Today.

[48] Tian Y., Shi C., Sun Y., Zhu C., Sun C.C., Mao S. (2015). Designing micellar Nanocarriers with improved drug loading and stability based on solubility parameter. Mol. Pharm..

[49] Lipinski C.A. (2000). Drug-like properties and the causes of poor solubility and poor permeability. J. Pharmacol. Toxicol. Methods.

[50] Da Silva F.L.O., Marques M.B.D.F., Kato K.C., Carneiro G. (2020). Nanonization techniques to overcome poor water-solubility with drugs. Expert Opin. Drug Discov..

[51] Das B., Baidya A.T.K., Mathew A.T., Yadav A.K., Kumar R. (2022). Structural modification aimed for improving solubility of lead compounds in early phase drug discovery. Bioorg. Med. Chem..

[52] Bergström C.A.S., Avdeef A. (2019). Perspectives in solubility measurement and interpretation. ADMET DMPK.

[53] Black S., Dang L., Liu C., Wei H. (2013). On the measurement of solubility. Org. Process Res. Dev..

[54] Boobier S., Hose D.R.J., Blacker A.J., Nguyen B.N. (2020). Machine learning with physicochemical relationships: Solubility prediction in organic solvents and water. Nat. Commun..

[55] Lovrić M., Pavlović K., Žuvela P., Spataru A., Lučić B., Kern R., Wong M.W. (2021). Machine learning in prediction of intrinsic aqueous solubility of drug-like compounds: Generalization, complexity, or predictive ability?. J. Chemom..

[56] Bhalani D.V., Nutan B., Kumar A., Singh Chandel A.K. (2022). Bioavailability Enhancement Techniques for Poorly Aqueous Soluble Drugs and Therapeutics. Biomedicines.

[57] Manallack D.T., Yuriev E., Chalmers D.K. (2018). The influence and manipulation of acid/base properties in drug discovery. Drug Discov. Today Technol..

[58] Merisko-Liversidge E., Liversidge G.G. (2011). Nanosizing for oral and parenteral drug delivery: A perspective on formulating poorly-water soluble compounds using wet media milling technology. Adv. Drug Deliv. Rev..

[59] Brewster M.E., Loftsson T. (2007). Cyclodextrins as pharmaceutical solubilizers. Adv. Drug Deliv. Rev..

[60] Korn C., Balbach S. (2014). Compound selection for development—Is salt formation the ultimate answer? Experiences with an extended concept of the “100mg approach”. Eur. J. Pharm. Sci..

[61] Seedher N., Kanojia M. (2009). Co-solvent solubilization of some poorly-soluble antidiabetic drugs. Pharm. Dev. Technol..

[62] Hahnenkamp I., Graubner G., Gmehling J. (2010). Measurement and prediction of solubilities of active pharmaceutical ingredients. Int. J. Pharm..

[63] Abraham M.H., Smith R.E., Luchtefeld R., Boorem A.J., Lou R., Acree W.E. (2010). Prediction of solubility of drugs and other compounds in organic solvents. J. Pharm. Sci..

[64] Hewitt M., Cronin M.T.D., Enoch S.J., Madden J.C., Roberts D.W., Dearden J.C. (2009). In silico prediction of aqueous solubility: The solubility challenge. J. Chem. Inf. Model..

[65] Lenoir D., Schramm K.W., Lalah J.O. (2020). Green Chemistry: Some important forerunners and current issues. Sustain. Chem. Pharm..

[66] Kopach M., Leahy D., Manley J. (2012). The green chemistry approach to pharma manufacturing. Innov. Pharm. Technol..

[67] Becker J., Manske C., Randl S. (2022). Green chemistry and sustainability metrics in the pharmaceutical manufacturing sector. Curr. Opin. Green Sustain. Chem..

[68] Mishra M., Sharma M., Dubey R., Kumari P., Ranjan V., Pandey J. (2021). Green synthesis interventions of pharmaceutical industries for sustainable development. Curr. Res. Green Sustain. Chem..

[69] DeSimone J.M. (2002). Practical approaches to green solvents. Science.

[70] Häckl K., Kunz W. (2018). Some aspects of green solvents. Comptes Rendus Chim..

[71] Santana-Mayor Á., Rodríguez-Ramos R., Herrera-Herrera A.V., Socas-Rodríguez B., Rodríguez-Delgado M.Á. (2021). Deep eutectic solvents. The new generation of green solvents in analytical chemistry. TrAC Trends Anal. Chem..

[72] Vanda H., Dai Y., Wilson E.G., Verpoorte R., Choi Y.H. (2018). Green solvents from ionic liquids and deep eutectic solvents to natural deep eutectic solvents. Comptes Rendus Chim..

[73] Omar K.A., Sadeghi R. (2022). Physicochemical properties of deep eutectic solvents: A review. J. Mol. Liq..

[74] Paiva A., Craveiro R., Aroso I., Martins M., Reis R.L., Duarte A.R.C. (2014). Natural Deep Eutectic Solvents—Solvents for the 21st Century. ACS Sustain. Chem. Eng..

[75] Espino M., de los Ángeles Fernández M., Gomez F.J.V., Silva M.F. (2016). Natural designer solvents for greening analytical chemistry. TrAC Trends Anal. Chem..

[76] Xu G., Shi M., Zhang P., Tu Z., Hu X., Zhang X., Wu Y. (2022). Tuning the composition of deep eutectic solvents consisting of tetrabutylammonium chloride and n-decanoic acid for adjustable separation of ethylene and ethane. Sep. Purif. Technol..

[77] Cao Y., Tao X., Jiang S., Gao N., Sun Z. (2020). Tuning thermodynamic properties of deep eutectic solvents for achieving highly efficient photothermal sensor. J. Mol. Liq..

[78] Pedro S.N., Freire C.S.R., Silvestre A.J.D., Freire M.G. (2021). Deep Eutectic Solvents and Pharmaceuticals. Encyclopedia.

[79] Cysewski P., Jeliński T., Przybyłek M. (2024). Experimental and Theoretical Insights into the Intermolecular Interactions in Saturated Systems of Dapsone in Conventional and Deep Eutectic Solvents. Molecules.

[80] Bazzo G.C., Pezzini B.R., Stulzer H.K. (2020). Eutectic mixtures as an approach to enhance solubility, dissolution rate and oral bioavailability of poorly water-soluble drugs. Int. J. Pharm..

[81] Kapre S., Palakurthi S.S., Jain A., Palakurthi S. (2024). DES-igning the future of drug delivery: A journey from fundamentals to drug delivery applications. J. Mol. Liq..

[82] Jeliński T., Przybyłek M., Mianowana M., Misiak K., Cysewski P. (2024). Deep Eutectic Solvents as Agents for Improving the Solubility of Edaravone: Experimental and Theoretical Considerations. Molecules.

[83] Duarte A.R.C., Ferreira A.S.D., Barreiros S., Cabrita E., Reis R.L., Paiva A. (2017). A comparison between pure active pharmaceutical ingredients and therapeutic deep eutectic solvents: Solubility and permeability studies. Eur. J. Pharm. Biopharm..

[84] Nguyen C.-H., Augis L., Fourmentin S., Barratt G., Legrand F.-X. (2021). Deep Eutectic Solvents for Innovative Pharmaceutical Formulations.

[85] Liu Y., Wu Y., Liu J., Wang W., Yang Q., Yang G. (2022). Deep eutectic solvents: Recent advances in fabrication approaches and pharmaceutical applications. Int. J. Pharm..

[86] García-Roldán A., Piriou L., Jauregi P. (2023). Natural deep eutectic solvents as a green extraction of polyphenols from spent coffee ground with enhanced bioactivities. Front. Plant Sci..

[87] Cysewski P., Przybyłek M., Jeliński T. (2024). Predicting sulfanilamide solubility in the binary mixtures using a reference solvent approach. Polym. Med..

[88] Hammond O.S., Bowron D.T., Edler K.J., Hammond S., Edler K.J., Bowron D.T. (2017). The Effect of Water upon Deep Eutectic Solvent Nanostructure: An Unusual Transition from Ionic Mixture to Aqueous Solution. Angew. Chem. Int. Ed..

[89] Gabriele F., Chiarini M., Germani R., Tiecco M., Spreti N. (2019). Effect of water addition on choline chloride/glycol deep eutectic solvents: Characterization of their structural and physicochemical properties. J. Mol. Liq..

[90] Mota F.L., Queimada A.J., Pinho S.P., Macedo E.A. (2008). Aqueous solubility of some natural phenolic compounds. Ind. Eng. Chem. Res..

[91] Shakeel F., Salem-Bekhit M.M., Haq N., Siddiqui N.A. (2017). Solubility and thermodynamics of ferulic acid in different neat solvents: Measurement, correlation and molecular interactions. J. Mol. Liq..

[92] Manic M.S., Villanueva D., Fornari T., Queimada A.J., MacEdo E.A., Najdanovic-Visak V. (2012). Solubility of high-value compounds in ethyl lactate: Measurements and modeling. J. Chem. Thermodyn..

[93] Haq N., Siddiqui N.A., Shakeel F. (2017). Solubility and molecular interactions of ferulic acid in various (isopropanol + water) mixtures. J. Pharm. Pharmacol..

[94] Vilas-Boas S.M., Alves R.S., Brandão P., Campos L.M.A., Coutinho J.A.P., Pinho S.P., Ferreira O. (2020). Solid-liquid phase equilibrium of trans-cinnamic acid, p-coumaric acid and ferulic acid in water and organic solvents: Experimental and modelling studies. Fluid Phase Equilib..

[95] Przybyłek M., Recki Ł., Mroczyńska K., Jeliński T., Cysewski P. (2019). Experimental and theoretical solubility advantage screening of bi-component solid curcumin formulations. J. Drug Deliv. Sci. Technol..

[96] Jeliński T., Przybyłek M., Cysewski P. (2019). Solubility advantage of sulfanilamide and sulfacetamide in natural deep eutectic systems: Experimental and theoretical investigations. Drug Dev. Ind. Pharm..

[97] Jeliński T., Stasiak D., Kosmalski T., Cysewski P. (2021). Experimental and theoretical study on theobromine solubility enhancement in binary aqueous solutions and ternary designed solvents. Pharmaceutics.

[98] Cysewski P., Jeliński T., Przybyłek M. (2022). Application of COSMO-RS-DARE as a Tool for Testing Consistency of Solubility Data: Case of Coumarin in Neat Alcohols. Molecules.

[99] Dassault Systèmes (2022). COSMOconf, Version 24.0.0, Dassault Systèmes.

[100] (2022). COSMOtherm, Version 24.0.0, Dassault Systèmes.

[101] Cysewski P., Jeliński T., Przybyłek M. (2023). Intermolecular Interactions of Edaravone in Aqueous Solutions of Ethaline and Glyceline Inferred from Experiments and Quantum Chemistry Computations. Molecules.

[102] Jeliński T., Przybyłek M., Różalski R., Cysewski P. (2024). Solubility of dapsone in deep eutectic solvents: Experimental analysis, molecular insights and machine learning predictions. Polym. Med..

[103] Cysewski P., Jeliński T., Przybyłek M., Mai A., Kułak J. (2024). Experimental and Machine-Learning-Assisted Design of Pharmaceutically Acceptable Deep Eutectic Solvents for the Solubility Improvement of Non-Selective COX Inhibitors Ibuprofen and Ketoprofen. Molecules.

[104] Jeliński T., Cysewski P. (2022). Quantification of Caffeine Interactions in Choline Chloride Natural Deep Eutectic Solvents: Solubility Measurements and COSMO-RS-DARE Interpretation. Int. J. Mol. Sci..

[105] Witte J., Neaton J.B., Head-Gordon M. (2017). Effective empirical corrections for basis set superposition error in the def2-SVPD basis: gCP and DFT-C. J. Chem. Phys..

[106] TURBOMOLE GmbH (2023). TURBOMOLE, Version 7.8.

[107] Bitencourt R.G., Cabral F.A., Meirelles A.J.A. (2016). Ferulic acid solubility in supercritical carbon dioxide, ethanol and water mixtures. J. Chem. Thermodyn..

[108] Cysewski P., Jeliński T., Przybyłek M. (2023). Finding the Right Solvent: A Novel Screening Protocol for Identifying Environmentally Friendly and Cost-Effective Options for Benzenesulfonamide. Molecules.

[109] Klamt A., Schüürmann G. (1993). COSMO: A new approach to dielectric screening in solvents with explicit expressions for the screening energy and its gradient. J. Chem. Soc. Perkin Trans..

[110] Acree W., Chickos J.S. (2017). Phase Transition Enthalpy Measurements of Organic and Organometallic Compounds and Ionic Liquids. Sublimation, Vaporization, and Fusion Enthalpies from 1880 to 2015. Part 2. C11–C192. J. Phys. Chem. Ref. Data.

